# High Concordance Between SYBR Green and TaqMan PCR for SARS-CoV-2 Detection in Nasopharyngeal and Saliva Samples

**DOI:** 10.3390/v17081130

**Published:** 2025-08-18

**Authors:** Muhareva Raekiansyah, Ratika Rahmasari, Fathan Baihaqy, Muhamad Irhamsyah, Nurul Izza Fajriani, Mila Meilani Putri, Botefilia Maharani, Rani Sauriasari, Takeshi Urano, Mya Myat Ngwe Tun, Kouichi Morita

**Affiliations:** 1 Department of Tropical Viral Vaccine Development, Institute of Tropical Medicine, Nagasaki University, Nagasaki 852-8523, Japan; m.raekiansyah@nagasaki-u.ac.jp (M.R.); moritak@nagasaki-u.ac.jp (K.M.); 2 Microbiology and Biotechnology Laboratory, Faculty of Pharmacy, Universitas Indonesia, Depok 16424, West Java, Indonesia; milamputri.mmp@gmail.com (M.M.P.); botefilia.maharani@gmail.com (B.M.); 3 Helix Laboratory, Depok 16431, West Java, Indonesia; fathan2000b@gmail.com (F.B.); muhammadirhamsyah@gmail.com (M.I.); 4 Department of Microbiology, School of Life Sciences & Technology, Institut Teknologi Bandung, Bandung 40132, West Java, Indonesia; izzafajrianimudatsir@gmail.com; 5 Clinical Pharmacy and Social Pharmacy Laboratory, Faculty of Pharmacy, Universitas Indonesia, Depok 16424, West Java, Indonesia; rani@farmasi.ui.ac.id; 6 Center for Vaccines and Therapeutic Antibodies for Emerging Infectious Diseases, Shimane University, Izumo 693-8501, Japan; turano@med.shimane-u.ac.jp; 7 Department of Virology, Institute of Tropical Medicine, Nagasaki University, Nagasaki 852-8523, Japan; 8 DEJIMA Infectious Disease Research Alliance, Nagasaki University, Nagasaki 852-8523, Japan

**Keywords:** SARS-CoV-2, COVID-19, SYBR green, RT-qPCR, naso-oropharyngeal swabs, saliva

## Abstract

During the COVID-19 pandemic, the standard diagnostic assay for SARS-CoV-2 detection was RT-qPCR using TaqMan probes, with samples primarily taken through nasal and oropharyngeal swabs. The TaqMan-based method is costly, highlighting the need for a more affordable alternative for SARS-CoV-2 diagnosis. As an alternative strategy, we developed and evaluated a SYBR Green-based RT-qPCR method targeting the RNA-dependent RNA polymerase (RdRp) gene of SARS-CoV-2. Under optimized RT-qPCR conditions, the sensitivity and linearity of the SYBR assays were assessed by using in vitro-transcribed RNA and RNA extracted from cultured SARS-CoV-2 isolates of the Wuhan reference strain and various circulating variants. Our results demonstrated that the SYBR Green-based RT-qPCR method was successfully developed with sufficient performance. The assay could detect up to 25 copies of in vitro-transcript RNA per reaction. Meanwhile, using the RNA extracted from cultured virus, the SYBR green assay was able to detect virus concentrations at least as low as 1 PFU/mL per reaction for all the variants tested. When tested on clinically relevant samples (88 naso-oropharyngeal swabs and 47 saliva samples), comparable results with the TaqMan assay were demonstrated. The Ct values of both methods for the positively detected samples were similar, with a difference in Ct of 0.72 ± 0.83 (*p* = 0.392) and −0.7765 ± 0.6107 (*p* = 0.209) for naso-oropharyngeal swab and saliva samples, respectively. These findings suggest that the SYBR method is reliable and thus offers an alternative assay for the detection of SARS-CoV-2. In particular, using saliva specimens could allow this assay to serve as a simple approach for SARS-CoV-2 detection.

## 1. Introduction

The coronavirus disease 2019 (COVID-19) pandemic, caused by severe acute respiratory syndrome coronavirus 2 (SARS-CoV-2), has created a global public health crisis over the past four years. During the peak of the pandemic in 2021–2022, health laboratories faced unprecedented challenges in conducting virologic testing as part of diagnostic procedures [1]. A lesson learned from the COVID-19 pandemic is that early detection and isolation of suspected individuals play a pivotal role in virus containment [2,3]. This underscores the importance of implementing extensive screening, particularly in affected areas. To address these needs, robust testing for widespread use in diverse laboratory settings is essential.

Since the beginning of the pandemic, SARS-CoV-2 detection has primarily relied on TaqMan-based reverse transcription-quantitative polymerase chain reaction (RT-qPCR) assays. This method was recommended by the World Health Organization (WHO) as the gold standard method for virus detection, using samples mainly taken by smear (swab) from the nasal and oropharyngeal areas of suspected individuals [4]. While highly sensitive and specific, TaqMan-based methods are expensive as they require specific fluorogenic probes, making diagnosis on a large scale difficult to perform, particularly in low- and middle-income countries. In addition, most of the protocols target multiple genes with different probes [4,5,6], which further increases the cost. From the experience of COVID-19, the enormous scale of the pandemic created shortages of reagents and testing materials, impacting the ability to meet global demand for diagnostic testing [7]. All these challenges highlight the need for more accessible and cost-effective diagnostic tests as alternatives to SARS-CoV-2 detection. To address this, RT-qPCR that utilizes SYBR Green chemistry could offer an alternative method that is more affordable and accessible.

The SYBR Green or TaqMan techniques are routinely utilized in real-time PCR for the detection and amplification of nucleic acids to confirm virus infection and other types of diseases. Due to its simple design, easy configuration, and low cost, the SYBR Green-based detection methodology is predominantly used. However, SYBR Green has its limitations. Unlike the TaqMan method, which uses specific oligonucleotide probe sequences that make it highly sensitive and specific, SYBR Green dye binds to any double-stranded DNA to emit a fluorescent signal [8]. Consequently, it can bind to non-specific products such as primer dimers or off-target amplicons, producing a false-positive signal. Nevertheless, despite the potential for non-target sequences to produce signals due to unspecific amplification, the accuracy of the SYBR Green-based method can be verified through melting curve analysis at the end of PCR. Differences in melting temperatures (Tm) allow for differentiation between target and non-target sequences [9]. Given these characteristics, the success of an RT-PCR assay using SYBR Green heavily depends on the careful selection of specific primers through in silico validation, followed by optimization of RT-PCR parameters.

SARS-CoV-2 is a single positive-stranded RNA virus with a genome approximately 29.9 kb in length. The SARS-CoV-2 genome is read through 16 ORFs: 2 for non-structural proteins, 4 for structural proteins, and 10 for accessory proteins [10,11]. Among all the open reading frames (ORFs), ORF1ab is the longest. It comprises two-thirds of the SARS-CoV-2 viral genome, making it an ideal target for nucleic acid-based detection. Specifically, the RNA-dependent RNA polymerase (RdRp) encoded by ORF1ab is an important target for the detection of SARS-CoV-2 by RT-qPCR due to its high specificity and superior sensitivity compared to other target genes [5,12]. In addition, the RNA-dependent RNA polymerase (RdRp) gene of SARS-CoV-2 is highly conserved and exhibits minimal sequence variation across different variants. This makes it a reliable target for molecular detection assays regardless of viral variant [13,14].

Previously, SYBR Green-based RT-qPCR assays have been described for the detection of SARS-CoV-2 using nasal and oropharyngeal samples [15,16,17,18,19,20,21]. However, studies reporting the use of SYBR Green assays with saliva specimens or validating their protocols using SARS-CoV-2 virus and its variants are very limited. Therefore, in this study, we developed a new SYBR Green-based assay targeting the RdRp gene for SARS-CoV-2 detection and validated its performance using viral RNA extracted from cultured SARS-CoV-2 isolates of the Wuhan reference strain and various circulating variants. The diagnostic value of the SYBR Green assay was then assessed by using naso-oropharyngeal and saliva samples, with a direct comparison to the TaqMan-based assay.

## 2. Materials and Methods

### 2.1. In Silico Primer Study

Primers were designed within a conserved region from SARS-CoV-2 RNA-dependent RNA polymerase (RdRp), with the sequence retrieved from an Indonesian patient isolate available in NCBI Genbank (Accession No. MZ026854.1). The sequence was submitted to online Primer-BLAST [22] to design primer pairs. The default parameters were maintained to ensure the reliability of the designed primer pair features. The specificity check was performed using the complete Refseq RNA database for Homo sapiens (taxid: 9606), Bacteria (taxid: 2), Fungi (taxid: 4751), and Apicomplexa (taxid: 5794). From the Primer-BLAST output, the Gibbs free energy (ΔG) and coverage rate across various SARS-CoV-2 strains (2000 genome dataset) were assessed using MFE primer 3.3 analysis [23]. Based on the ΔG scores, the best primer pairs were selected from optimal candidates for subsequent in vitro validation. For further analysis, the secondary structures of the primer-probe binding sites for the selected primer candidates in the SARS-CoV-2 RdRp gene were examined. The minimum free energy structures were predicted using the RNAstructure 6.4 [24] software package. Visualization of RNA secondary structure was created with VARNA 3.93 [25] and refined using additional graphical tools.

### 2.2. Samples and Ethical Clearance

This study utilized de-identified nasal and oropharyngeal VTM samples obtained during routine diagnostic procedures. The samples from positive and negative cases were randomly chosen from our laboratory’s collection. Similarly, for saliva samples of positive and negative groups, the residual de-identified specimens collected in phosphate-buffered saline (PBS) were randomly selected.

### 2.3. Virus Propagation and Titration

The SARS-CoV-2 Wuhan strain and its variants Alpha, Beta, Delta, and Omicron BA.1, provided by the National Institute of Infectious Disease, Japan, were propagated in Vero-E6 cells (African green monkey kidney cells) using minimum essential medium (MEM) supplemented with 10% fetal calf serum (FCS). Five days after infection, the supernatant was harvested, centrifuged at 2500 rpm for 10 min, and stored at −80 °C as virus stock. For virus quantification via plaque assay, Vero TM cells were used with the same culture medium. Briefly, confluent Vero TM cells in 24-well plates were inoculated in duplicate wells with 200 µL/well of tenfold serial dilutions of virus stock. After 1.5 h of viral adsorption, 0.5 mL of 1.25% methylcellulose 4000 in 2% FCS MEM was added to each well. The plates were incubated at 37 °C with 5% CO_2_ for 5 days. To visualize plaques, the culture medium was removed, and the cells were fixed overnight with 10% formaldehyde, followed by staining with a 1% crystal violet solution. The viral plaques were then counted. All experiments involving infectious SARS-CoV-2 were conducted in a biosafety level 3 (BSL-3) laboratory at Nagasaki University, following established BSL-3 safety protocols.

### 2.4. Total Nucleic Acid Extraction

A total of 300 µL of viral transport medium (VTM) from nasal and oropharyngeal swabs was used for total nucleic acid extraction. RNA extraction was carried out using the TANBead Smart LabAssist-32 system (Taiwan Advanced Nanotech Inc., Taoyuan City, Taiwan) following the manufacturer’s instructions. Each sample was eluted in a final volume of 100 µL, and the extracted nucleic acid was stored at −80 °C until further use. For RNA extraction of virus and saliva samples, 100 µL of infected cell supernatant or saliva was processed using the Nextractor NX-48 robotic system and the NX-48S Viral NA Kit (Genolution Inc., Seoul, Republic of Korea) according to the manufacturer’s instructions. The final elution was carried out with 100 µL of elution buffer.

### 2.5. Generation of In Vitro-Transcribed RNA

A positive control RNA standard was generated by cloning the targeted RdRP region of the SARS-CoV-2 genome into the pBluescript II KS (-) vector (Genscript, Piscataway, NJ, USA) using SmaI and XhoI restriction sites. Briefly, the positive clone was cultured overnight in LB broth containing 50 μg/mL ampicillin. Plasmid DNA was extracted using the GeneJET Plasmid Miniprep Kit (Thermo Scientific, Waltham, MA, USA) and quantified by spectrophotometric analysis (NanoDrop, Thermo Scientific, Waltham, MA, USA). In vitro transcription was performed using the TranscriptAid T7 High Yield Transcription Kit (Thermo Scientific, Waltham, MA, USA) following the manufacturer’s instructions. The in vitro transcribed RNA was then purified, and RNA yield was measured using NanoDrop. The RNA copy number per microliter was calculated using the formula: (NA × C)/MW, where NA is Avogadro’s constant (mol^−1^), C is the concentration (g/μL), and MW is the molecular weight (g/mol).

### 2.6. TaqMan- and SYBR Green-Based RT-qPCR Assays for SARS-CoV-2 Detection

For the TaqMan-based assay used to compare with the SYBR Green assay on nasal and oropharyngeal samples, a commercial 2019-nCoV Nucleic Acid Diagnostic Kit (Sansure Biotech, Changsha, China) was employed. This kit detects the ORF1ab and N genes of SARS-CoV-2, as well as the human RNase P gene for internal control. Results were considered positive if the Ct values for both the ORF1ab and N genes were ≤40; if these values were >40, the results were deemed negative. The assay was performed following the manufacturer’s instructions. Each 20 µL reaction contained 9 µL of master mix (enzyme mix + 5× buffer for real-time PCR), 1 µL of primer, 5 µL of nuclease-free water, and 5 µL of RNA. The thermal cycling conditions included reverse transcription at 50 °C for 20 min, followed by reverse transcriptase inactivation at 95 °C for 3 min, and then 40 cycles at 94 °C for 10 s and 55 °C for 40 s. For comparison with the SYBR Green assay on saliva samples, an in-house TaqMan-based assay was employed as described previously. Briefly, 5 μL of RNA was used for RT-qPCR, and amplification of the nucleocapsid (N) gene was carried out in a 20 µL reaction mixture. The mixture consisted of 5 μL of TaqMan master mix, 7 µL of nuclease-free water, 1 µL each of 0.5 µM forward and reverse primers, 0.25 µM probe, SARS-CoV-2 N-gene-specific primers, and TaqMan Fast Virus 1-Step Master Mix (Life Technologies, Carlsbad, CA, USA) [26]. The primers and probes for SARS-CoV-2 were described previously [27]. The SYBR Green-based one-step RT-qPCR assay targeting the RdRp gene was conducted using the iTaq Universal SYBR Green Super-mix (Bio-Rad, Hercules, CA, USA) according to the manufacturer’s instructions. Each 10 μL reaction included 5 μL of iTaq Universal SYBR Green Supermix (2×), 0.125 μL of iScript reverse transcriptase, 1 μL of forward and reverse primers, 1.875 μL of nuclease-free water, and 2 μL of RNA template. In preliminary experiments, the primer concentrations were adjusted to achieve optimal assay conditions. Thermal cycling was run with the following cycle parameters: 50 °C for 10 min for reverse transcription, 95 °C for 1 min for initial denaturation, and then 40 cycles at 95 °C for 10 s and 60 °C for 30 s. This was followed by melting curve analysis, during which fluorescence signals from the amplified products were continuously recorded as the temperature increased from 65 °C to 95 °C, with data collected at 0.3 °C intervals. Thermal cycling was performed on a QuantStudio 6 Pro real-time PCR system (Thermo Fisher Scientific, Waltham, MA, USA). The PCR runs were analyzed using QuantStudio™ Real-Time PCR Software V1.7.2.

### 2.7. Analytical Sensitivity of SYBR Green-Based Assay

The analytical sensitivity of the SYBR Green-based assay was evaluated by using tenfold serial dilutions of in vitro-transcribed RNA and fivefold serial dilutions of RNA extracted from infected culture fluid of the SARS-CoV-2 Wuhan strain and its variants. Each diluted RNA standard was subjected to RT-qPCR in triplicate. For in vitro-transcribed RNA, calibration curves were plotted as Ct value versus the logarithm of RNA copy number per reaction. For the SARS-CoV-2 Wuhan strain and its variants, calibration curves were represented as Ct value versus plaque-forming units (PFU) per milliliter per reaction. The amplification efficiency (E) was calculated using the formula: E = 100 × (10^−1/s^ − 1), where s is the slope of the calibration curve.

### 2.8. Gel Electrophoresis

The size of the RT-qPCR products was verified by agarose gel electrophoresis. Amplified products were separated in a 2% agarose gel containing 0.01% *v*/*v* GelRed (Biotium, Fermont, CA, USA) at 100 V for 40 min. The gel was subsequently visualized with a UV transilluminator. The expected sizes of the amplified RdRp gene fragments ranged from 110 to 115 bp.

### 2.9. Intra- and Inter-Assays

To assess the reproducibility and repeatability of the SYBR green assays, both intra-and inter-assays were conducted in three clinical samples with varying Ct values. For the intra-assay evaluation, three replicates were performed on the same PCR plate. For the inter-assay evaluation, the test was repeated across three different PCR plates under identical conditions. The mean, standard deviation (SD), and coefficient of variation (CV) were calculated based on the Ct values obtained. According to the bioanalytical method validation guidelines, CV values of less than 10% for intra-assay and 15% for inter-assay tests were considered acceptable.

### 2.10. Statistical Analysis

The cycle threshold (Ct) values for the amplification of each target gene were analyzed. Statistical differences between groups were assessed using one-way analysis of variance (ANOVA), with a significance level set at *p* < 0.05.

## 3. Results

### 3.1. In Silico Study

**Figure 1:**
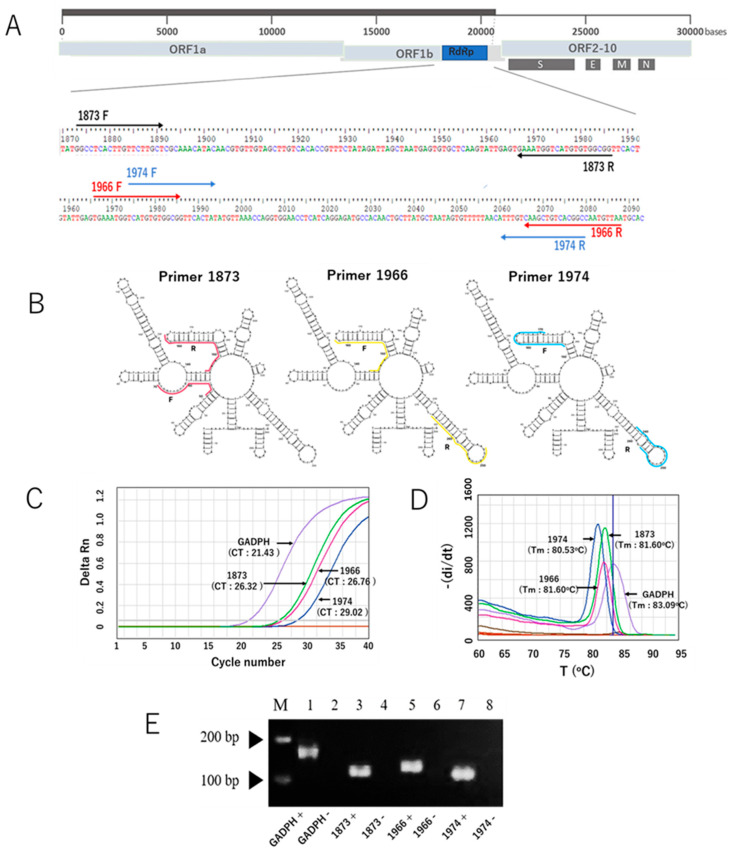
The three RdRp primer pairs and their secondary structures and characteristic performance in the SYBR Green-based RT-qPCR assay for the detection of SARS-CoV-2. (**A**) The three RdRp primer pairs with respect to their location in the RdRp gene and the whole genome. Primers 1873, 1966, and 1974 are indicated in light brown, red, and blue colors, respectively. F represents the forward primer, and R represents the reverse complementary sequence of the reverse primer. (**B**) Visualization of RNA secondary structure of the RdRp gene fragments amplified using the forward and reverse primers. (**C**) Representative amplification curves of the SYBR green assays with the three different RdRp primers and GADPH gene as a control primer pair, using in vitro-transcribed RNA. (**D**) Melting curves of the products amplified with the three RdRp primers and GADPH gene. (**E**) Distinct PCR products amplified with three different primers and the GAPDH gene (lines 1, 3, 5, and 7) were visualized on a 2% agarose gel. The PCR product sizes for the respective primers are 170 bp (GAPDH), 114 bp (1873), 123 bp (1966), and 109 bp (1974). Lines 2, 4, 6, and 8 indicate negative control of the assays (nuclease-free water). Note: the arrows mark the bands with molecular weights of 100 bp and 200 bp.

After our in silico study to develop a new SYBR Green-based RT-qPCR assay, we initially selected ten primer pairs targeting the RdRp gene of SARS-CoV-2. The selection was based on Gibbs free energy (ΔG) scores, along with other parameters such as melting temperature (Tm) and GC content. Of the ten, three primer sets had the most negative ΔG values and melting temperatures of approximately 59 °C. These primer sets were identified as RdRp 1873, RdRp 1966, and RdRp 1974, which were subsequently validated through in vitro experiments. The sequences of the primers are listed in Table 1, while the locations of the RdRp-specific primers in the SARS-CoV-2 genome are shown in Figure 1A. The RNA secondary structure of the target sequences was predicted and analyzed using the minimum free energy method. The binding positions of each primer are indicated in Figure 1B, and the results showed that our primers predominantly bind to target sequences located within stem structures. Using the in vitro-transcribed RNA, we optimized the new SYBR Green-based RT-qPCR protocol by adjusting annealing temperature and primer concentration. The characteristic performance of each primer pair is shown in the generated amplification curve (Figure 1C). The human housekeeping gene Glyceraldehyde 3-phosphate dehydrogenase (GAPDH) was used to validate the primer design. Different primers produced different Ct values, and no unspecific signal was observed for any of the no-template controls. Melting curve analysis validated the amplification curve findings, which showed a distinct melt peak line (Figure 1D). This confirmed the production of a single PCR amplicon in each assay. Furthermore, the visualization of PCR products using agarose gel electrophoresis revealed single bands for each assay, with no bands appearing in the negative control samples (Figure 1E). Taken together, these results indicate that our newly developed SYBR Green assay with the newly designed primers specifically amplified the target product, as expected. In addition, because the RdRp 1873 primer generated the lowest Ct value compared to the other primers, indicative of greater amplification efficiency, we chose this primer for further evaluation.

### 3.2. The Sensitivity of SYBR Green Assay

**Figure 2:**
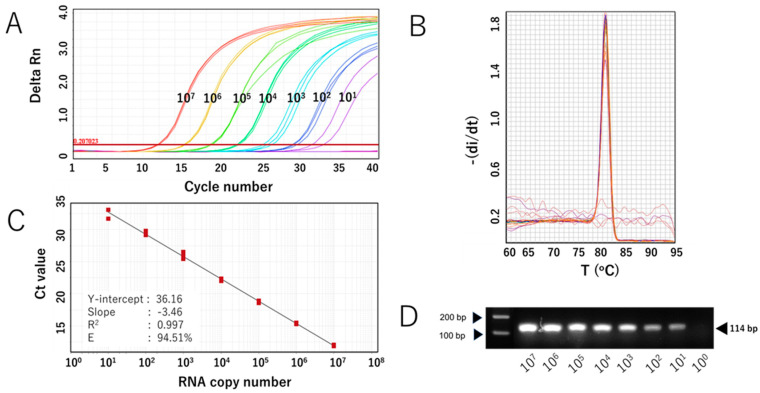
Analytical sensitivity of the SYBR Green-based RT-qPCR for SARS-CoV-2 detection (with RdRp 1873 as primer) using in vitro-transcribed RNA standards. (**A**) Representative amplification curve of RdRp 1873 assay using RNA standard copy numbers. Amplification plots refer to the cycle number versus fluorescence of serially diluted in vitro-transcribed RNA standards (copies/reaction). (**B**) Corresponding melting curves for amplified products. Unique single peaks with Tm at around 80.60 °C were generated in all positive samples. (**C**) Standard curves of the RdRp 1873 assay. RNA copy number (log starting quantity) was plotted against the mean cycle threshold (Ct) value. The coefficient of determination (R^2^) and the equation of the regression curve (Y) were calculated. (**D**) Representative RT-qPCR amplicons in a 2% agarose gel, showing a single band of 114 bp. Note: the arrows on the left side mark the bands with molecular weights of 100 bp and 200 bp.

The sensitivity of the SYBR Green-based assay with RdRp 1873 as primer (RdRp 1873 assay) was then evaluated. In vitro-transcribed RNA containing the target gene was serially diluted to concentrations ranging from 10^7^ to 10^1^ copies/μL and used for RT-qPCR. The assay was performed in triplicate. The RdRp 1873 assay successfully amplified the standard RNA down to 10^1^ copies/μL; however, one replicate at this concentration failed to amplify. No amplification was generated at 100 copies/μL (Figure 2A). Melting curve analysis again validated the amplification curve findings, showing a single peak with a Tm of approximately 81.0 °C in all samples (Figure 2B). When SYBR Green-based RT-qPCR products were verified through gel electrophoresis, single amplicons of the expected size were revealed (Figure 2C). Furthermore, the Ct value showed a linear correlation with the logarithm of RNA copy number, with an R^2^ = 0.997 (Figure 2B). The standard curve equation was Y = −3.46X + 36.16, and the amplification efficiency (E) was 94.51%. These parameters indicate that the RT-qPCR amplification of the RdRp 1873 assay is efficient [28]. Overall, the findings from the analytical sensitivity experiment using in vitro RNA standards demonstrate that our RdRp 1873 SYBR Green-based assay is sufficiently sensitive for detecting SARS-CoV-2.

**Figure 3:**
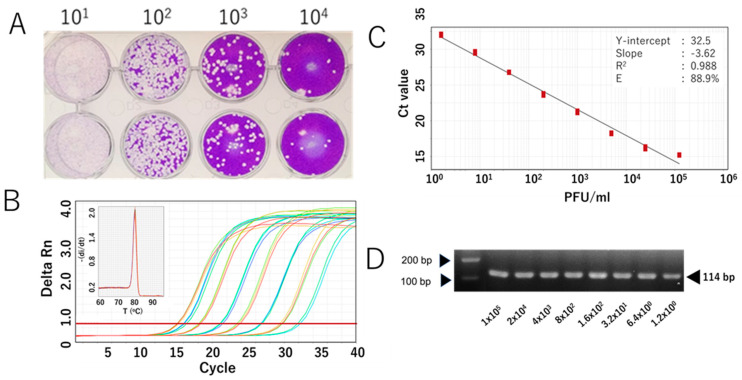
Analytical sensitivity of the RdRp 1873 assay, determined using the authentic SARS-CoV-2 Wuhan strain. (**A**) Representative plaque assay of the Wuhan strain in Vero-TM cells to determine virus titer. (**B**) Amplification curves of the amplicons of the original Wuhan strain (serially diluted fivefold) after RdRp 1873 assay. The corresponding melting peak is shown in the inset panel. (**C**) Standard curves generated by the RdRP 1873 assay using the Wuhan strain. Virus titer (PFU/mL, log starting quantity) is indicated and plotted against the mean cycle threshold (Ct) value. The coefficient of determination (R^2^) and the equation of the regression curve (Y) were calculated. (**D**) Representative RT-qPCR amplicons visualized in a 2% agarose gel, showing a single band of 114 bp. Note: the arrows on the left indicate bands with molecular weights of 100 bp and 200 bp.

**Figure 4:**
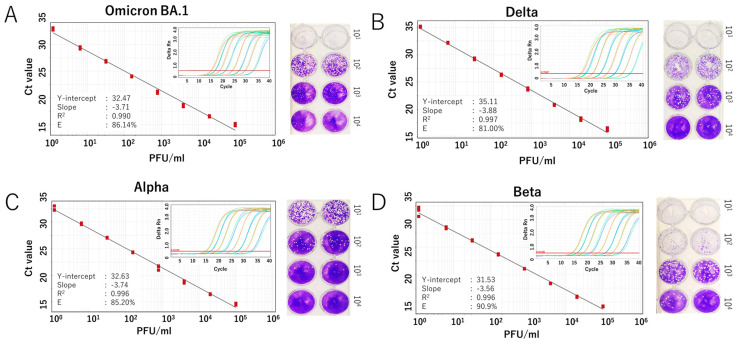
Analytical sensitivity of the SYBR Green-based RT-qPCR for detecting SARS-CoV-2 variants. Standard curves generated from fivefold serial dilutions of RNA extracted from infected culture fluid of the virus variants are shown for (**A**) Omicron BA.1 variant, (**B**) Delta variant, (**C**) Alpha variant, and (**D**) Beta variant. A representative plaque assay to determine the viral titer of each strain is shown in the right panel. The coefficient of determination (R^2^), Y-intercept, slope, and PCR efficiency (E) were calculated for each variant. Amplification curves for each variant are also displayed in the inset panel.

We further evaluated the RdRp 1873 assay using RNA extracted from cultured SARS-CoV-2 Wuhan strain. Additionally, since SARS-CoV-2 is prone to genetic evolution through mutation, we also assessed the newly developed SYBR Green assay in detecting various SARS-CoV-2 variants, including Omicron BA.1, Delta, Alpha, and Beta. By performing RT-qPCR with fivefold serial dilutions of RNA extracted from the viruses, the results demonstrated that the SYBR Green-based assay could reliably detect the presence of the virus at concentrations as low as 1 PFU/mL per reaction. For virus variants, a similar limit of detection to that of the original Wuhan strain was demonstrated. The amplification efficiency (E) ranged from 81.0% to 90.9% (Figure 3 and Figure 4). Although the PCR efficiency was lower for certain variants (e.g., 80% for Delta), it remained sufficient for detecting SARS-CoV-2 RNA. For the Wuhan strain, we could observe a single amplicon band after gel electrophoresis analysis, confirming specific and accurate amplification (Figure 3D).

### 3.3. Validation of the SYBR Green Assay Using Clinical Samples

**Figure 5:**
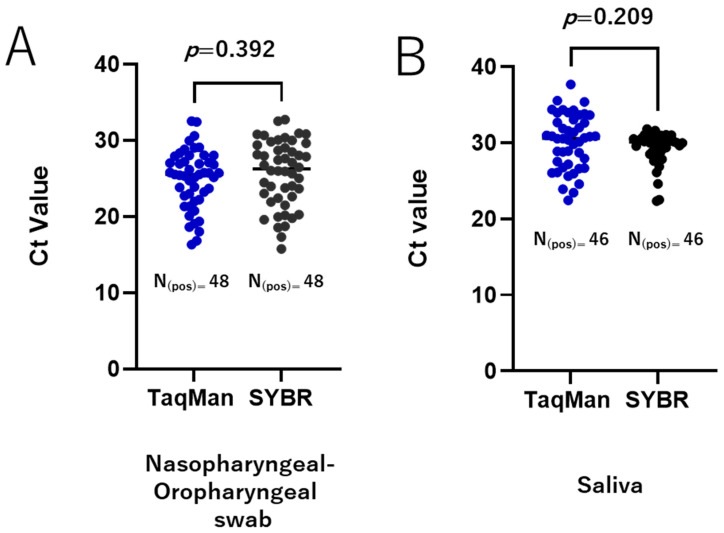
Validation of the SYBR Green-based RT-qPCR method in clinical samples. A total of 135 clinically relevant samples were tested, comprising 88 nasopharyngeal and oropharyngeal swab samples (48 positive and 40 negative) and 47 saliva samples (46 positive and 1 negative). Agreement between the TaqMan-based assay and the RdRp 1873 SYBR Green-based assay is shown for nasopharyngeal and oropharyngeal swab samples (**A**) and saliva samples (**B**). A *p*-value of <0.05 is considered statistically significant.

To validate our SYBR Green-based RT-qPCR protocols, we re-tested a set of clinically relevant samples, including nasopharyngeal and oropharyngeal swabs and saliva, which were qualitatively tested for SARS-CoV-2 during routine diagnostics. The performance of the SYBR Green-based methods was compared to that of the TaqMan-based assay. All positive samples identified by RT-qPCR using the TaqMan method were also detected as positive by the RdRp 1873 SYBR Green-based method for both the nasopharyngeal and oropharyngeal swabs and the saliva samples. Similarly, all negative samples were accurately identified as negative. For the positive samples, the Ct values obtained by the two methods were comparable with an average Ct difference of 0.72 ± 0.83 (*p* = 0.392) for nasopharyngeal and oropharyngeal swabs (Figure 5A) and 0.78 ± 0.61 (*p* = 0.209) for saliva samples (Figure 5B). These findings demonstrate a high agreement between the TaqMan-based assay and the RdRp 1873 SYBR Green-based assay.

### 3.4. Reproducibility and Repeatability of the SYBR Green Assay

Finally, we assessed the reproducibility and repeatability of our SYBR Green-based assays using naso-oropharyngeal samples with varying Ct values that were tested repeatedly. The inter-assay coefficient of variation (CV) was 3.84 ± 1.14, and the intra-assay CV was 2.79 ± 3.03 (Table 2). These levels of variability are considered acceptable, demonstrating that our SYBR Green-based RT-qPCR assay is reliable and reproducible.

## 4. Discussion

Accurate and affordable testing is an essential part of managing emerging infections. This is especially true for the SARS-CoV-2 pandemic, which has impacted hundreds of millions of people worldwide. Molecular techniques, primarily RT-qPCR, had proven successful in identifying the novel SARS-CoV-2 [29] and were later widely used during the pandemic to diagnose COVID-19 in the laboratory setting [30]. This method is highly sensitive and capable of detecting the virus in the early stages of infection [31].

The RT-qPCR-based assay for detecting SARS-CoV-2 was first developed at the Charité Institute of Virology in Germany [5], followed by other countries, including China and the United States [32]. All the assays used TaqMan probe and were designed to target the SARS-CoV-2 RdRp gene, along with the E and N genes [33]. Later, the World Health Organization (WHO) recognized TaqMan-based RT-qPCR as the gold standard for detecting SARS-CoV-2. As SARS-CoV-2 spread globally and viral sequence data rapidly expanded, efforts were made to develop RT-qPCR assays based on updated reference sequences to improve diagnostic accuracy.

The present study developed a SYBR Green-based assay with new primers targeting the RdRp gene of SARS-CoV-2. We selected three primers (RdRp 1873, RdRp 1966, and RdRp 1974) based on parameters such as ΔG scores, Tm, and GC% content. Gibbs free energy (ΔG) calculations predict primer dimer formation in PCR. It represents the amount of energy required for a primer to form a particular secondary structure with itself. These primer–primer interactions can competitively inhibit binding to target DNA, reduce the available primers in the reaction, and exhaust deoxynucleotides, leading to decreased amplification efficiency [34]. A more negative ΔG indicates a lower likelihood of primer dimer formation. Prediction of secondary structures of the RdRp fragments amplified by the forward and reverse primers showed that the three primers mostly bind to target sequences located on the stem structure. As a general principle, to ensure efficient and specific assay design, it is suggested to select primers from conserved regions located on stem structures [35]. This principle is especially relevant for SARS-CoV-2, which carries an RNA genome as its genetic material, making it highly susceptible to degradation by RNase enzymes [36]. The stem structure is more stable than the loops that are easily cleaved. Improper sample handling, such as inadequate storage conditions, can lead to RNA cleavage, which decreases detection sensitivity and may result in false-negative outcomes. Thus, by avoiding loops and targeting stem regions in the design of molecular detection assays for RNA viruses, the original RNA levels can be better preserved, even if the RNA is degraded due to improper sample handling [37]. However, these general suggestions still need to be verified through further in vitro experiments.

We chose primer RdRp 1873, which showed higher amplification efficiency than the other two primers, for further performance validation. The first approach used in vitro-transcribed RNA. The lack of an RNA standard from virus isolates or authentic virus is a significant barrier to implementing and validating RT-qPCR assays for SARS-CoV-2 detection. This challenge was particularly evident during the early pandemic when virus isolates were not available or when laboratories lacked high-containment facilities. To address this, generating in vitro-transcribed RNA from plasmids containing the target sequence provides an effective alternative. This approach can be applied to any pathogen as long as sequence data are available. It is fast, scalable, and capable of producing RNA in large quantities. Using this approach, we successfully generated an in vitro-transcribed RNA standard containing the target sequence and amplified it in 10-fold serial dilutions to estimate the minimum number of viral genome copies that could be detected. The results showed our RdRp 1873 assay could detect as few as 1 × 10^1^ copies/mL of the RNA transcribed standard, with a Ct value of around 34. This limit of detection is typical for RT-qPCR assays for viral RNA genome quantification, such as for SARS-CoV-2 [5,38]. In addition, our SYBR assay efficiently amplified the transcribed RNA template with an efficiency rate of approximately 95%, which was within the acceptable range for RT-qPCR assays [28].

The second approach to validate the performance of our SYBR assay involved using RNA extracted from cultured SARS-CoV-2. While in vitro-transcribed RNA only represents a simplified, synthetic version of the target RNA, the live virus reflects the full complexity of the viral particle, including the complete genome structure, which may affect PCR amplification. It also better mimics the behavior of an infectious virus or intact viral particles in clinical samples. Validation showed that the SYBR assay achieved a detection limit of 1.2 PFU/mL and an efficiency of approximately 90%, demonstrating robust performance for the Wuhan strain and all tested SARS-CoV-2 variants. The results also indicate that its detection capability is not affected by new mutations or emerging variants. Nonetheless, periodic reassessment of the assay is still necessary to ensure its reliability over time.

Last but not least, we evaluated the diagnostic value of our SYBR Green-based assay using clinically relevant samples. This validation step is crucial to ensure its applicability in real-world scenarios. For this purpose, we used two types of samples: nasopharyngeal and oropharyngeal swabs and saliva, which differed in their characteristics and matrix composition. These two sample types reflect common diagnostic practices in SARS-CoV-2 detection in clinical laboratory settings. Nasopharyngeal swabs are considered the gold standard, while saliva has emerged as a viable alternative sample. Direct comparison between our SYBR Green-based RT-qPCR assay and the TaqMan probe-based assay showed similar performance. There was a high level of agreement in Ct values between the two assays, indicating that the performance of our SYBR assay was reliable. Furthermore, our SYBR assay demonstrated high reproducibility, as indicated by both intra- and inter-assay results. This further confirms the robustness of the assay.

Previously, several studies developing RT-qPCR assays utilizing SYBR Green for SARS-CoV-2 detection validated the performance of their assays using nasal or oropharyngeal specimens [15,16,17,18,19,20,21]. To the best of our knowledge, there was only one report that established the SYBR green assay for the detection of SARS-CoV-2 using saliva samples [39], and this assay was used for community mass screening to identify asymptomatic infections. Our current study demonstrated comparable results between SYBR and the TaqMan assay, both in nasal-oropharyngeal specimens and saliva samples, suggesting that our SYBR method is reliable.

Utilizing saliva specimens for SARS-CoV-2 detection offers some advantages when compared to nasal-oropharyngeal swabs. It is easy to collect, non-invasive, and allows for self-collection without the need for professional assistance [40]. The diagnostic performance of saliva for SARS-CoV-2 molecular detection was systematically evaluated in meta-analysis studies. The results demonstrated that saliva and nasal-oropharyngeal swabs have comparable sensitivity [41,42,43], supporting the use of saliva as an alternative for COVID-19 diagnosis.

In the post-SARS-CoV-2 pandemic era, continuous surveillance is crucial to monitor the presence of the virus in the population and prevent potential future outbreaks. This is especially important in high-risk settings such as hospitals, nursing homes, and schools. Mass screening has proven to be an effective approach, as demonstrated during the last pandemic. In the context of large-scale testing, where the speed and frequency of testing are more important than test sensitivity [44,45], the SYBR Green-based assay provides an easier and more cost-effective method while still maintaining high sensitivity and accuracy in detecting the virus. In the context of Japan, according to our calculations, the overall reagent cost for the in-house SYBR method (including SYBR green master mix and primers) is approximately USD 0.44 per test, while the cost of the TaqMan probe (including TaqMan master mix, primers, and probe) is around USD 1.50 per test. These estimates do not include the cost of RNA extraction reagents or consumables. The cost of the SYBR Green method is even lower when using saliva samples [39]. However, it is important to note that these costs can vary significantly based on factors such as the specific supplier, brand, and the scale of the experiment. Nevertheless, the SYBR Green assay offers an attractive alternative to the TaqMan gold standard.

A key advancement of our study is the validation of a new SYBR Green-based assay for the detection of SARS-CoV-2 using RNA extracted from the cultured virus of Wuhan and the virus’s multiple variants. This study also highlights the applicability of the SYBR Green assay with saliva samples, offering a practical solution for large-scale SARS-CoV-2 testing. To ensure effective detection of the diverse SARS-CoV-2 variants circulating in Indonesia, we developed and optimized new primer sets for our SYBR Green assay targeting the RdRp gene, using a comprehensive dataset of gene sequences from Indonesian isolates. However, our study has certain limitations. The number of clinical samples analyzed was still limited, which may affect the broader applicability of the new SYBR Green assay. In addition, while our assay was validated using multiple SARS-CoV-2 variants, further validation is necessary to ensure its effectiveness against newly emerging variants. Future studies with larger and more diverse samples are needed to strengthen the diagnostic utility of the assay.

## 5. Conclusions

To conclude, we described a reliable method of a newly developed SYBR Green-based RT-qPCR assay targeting the RdRp gene for detecting SARS-CoV-2. It efficiently detected the original Wuhan strain and different SARS-CoV-2 variants. When tested on clinically relevant samples, the results were in high agreement with those obtained using the TaqMan-based RT-qPCR. This assay could serve as a simple approach for SARS-CoV-2 detection.

## Figures and Tables

**Table 1:** Three selected primers * generated in silico and evaluated in this study.

No	Primer	Sequence (5′>3′)	Length (bp)	Amplicon Length (bp)	Tm (°C)	GC%	ΔG (kcal/mol)	SARS-CoV (taxid:2901879)
1	Primer 1873	F GGCCTCACTTGTTCTTGCTC	20	114	59.12	55	−21.84	NC_004718.3 SARS
		R CCGCCACACATGACCATTTC	20		59.83	55	−22.26	
2	Primer 1966	F TGAAATGGTCATGTGTGGCG	20	123	59.12	50	−21.83	NC_004718.3 SARS
		R TTAACATTGGCCGTGACAGC	20		59.12	50	−21.83	
3	Primer 1974	F TCATGTGTGGCGGTTCACTA	20	109	59.32	50	−21.78	NC_004718.3 SARS r2
		R GCCGTGACAGCTTGACAAAT	20		59.41	50	−22.01	

* Result of comparison of 2000 genome sequences of SARS-CoV-2 from GenBank.

**Table 2:** Intra- and inter-reproducibility of SYBR Green-based assay.

Sample	Intra-Assay Variation ^a^		Inter-Assay Variation ^b^	
	Cycle Threshold (Ct) ^c^	CV (%)	Cycle Threshold (Ct) ^c^	CV (%)
Sample #1	20.39 ± 0.44	2.14	21.16 ± 1.07	5.04
Sample #2	21.78 ± 0.33	0.13	22.15 ± 0.61	2.77
Sample #3	33.56 ± 2.04	6.09	32.18 ± 1.20	3.72
				
Overall CV (%) ^c^	2.79 ± 3.03		3.84 ± 1.14	

^a^ Intra-assay variation was determined for the triplicate runs of clinical samples at different Ct values in the same PCR run. ^b^ Inter-assay variation was calculated based on values obtained in three separate PCR runs. ^c^ Mean ± standard deviation (SD) CV: coefficient of variation.

## Data Availability

The datasets generated and/or analyzed during the current study are available in the manuscript.
